# Crop establishment and diversification strategies for intensification of rice-based cropping systems in rice-fallow areas in Odisha

**DOI:** 10.1016/j.fcr.2023.109078

**Published:** 2023-10-15

**Authors:** Panneerselvam Peramaiyan, Amit Kumar Srivastava, Virender Kumar, Lavanya P. Seelan, Narayan Chandra Banik, Suryakanta Khandai, Nabakishore Parida, Vivek Kumar, Aurovinda Das, Sanghamitra Pattnaik, Dilip Ranjan Sarangi, Pavan Kumar Yeggina, Ashok Yadav, Andrew J. McDonald, Peter Craufurd, Sudhanshu Singh, Ram K. Malik

**Affiliations:** a International Rice Research Institute, New Delhi, India; b International Rice Research Institute, Los Baños, Philippines; c International Maize and Wheat Improvement Centre, NASC Complex, New Delhi, India; d Odisha University of Agriculture and Technology, Bhubaneshwar, India; e ICAR-National Rice Research Institute, Cuttack, India; f Soil and Crop Sciences Section, School of Integrative Plant Sciences, Cornell University, Ithaca, NY, USA; g International Maize and Wheat Improvement Centre, Kathmandu, Nepal

**Keywords:** Direct Seeded Rice, Mechanical Transplanted Rice, Sustainable intensification, Crop diversification, Rice-fallows, Suitability mapping

## Abstract

**Context or problem:**

In the Indian state of Odisha, rice-based system productivity is poor due to: (i) low rice yield in the monsoon (wet) season (2–4 t ha^−1^ compared to 6–8 t ha^−1^ in Punjab or Haryana); and (ii) limited cropping during the post-monsoon (dry) season (59% of the wet season rice area is left fallow in the dry season).

**Objective:**

Our study identifies strategies for increasing rice-based system productivity through: (i) alternative crop establishment methods in the wet season (Dry-Direct Seeded Rice or DSR, and mechanical puddled transplanted rice or PTR-M) to traditional methods such as broadcasting followed by post-emergence tillage (locally known as *beushening*) and manual random puddled transplanted rice (PTR-R); (ii) to identify rice-fallow areas suitable for pulse and oilseed cultivation in the dry season; and (iii) to evaluate the performance of short-duration pulses (green gram, *Vigna radiata*; black gram, *Vigna mungo*), and oilseeds (*Brassica rapa* var. toria, *Helianthus annuus*) in rice-fallow areas in the dry season.

**Methods:**

On-farm experiments were conducted between 2017 and 2019 in three districts of Odisha (Bhadrak, Cuttack and Mayurbhanj) to evaluate DSR compared to *beushening* and PTR-R; and PTR-M compared to PTR-R and manual line puddled transplanted rice (PTR-L) in the wet season. The data from Landsat-8 Operational Land Imager (OLI) and Sentinel-1satellite sensors was used to identify rice-fallow areas, and the daily SMAP (Soil Moisture Active Passive) L-band soil moisture was used for mapping suitable rice-fallow areas for growing pulses and oilseeds. Short duration crops were evaluated in suitable rice-fallow areas.

**Results:**

In the wet season, DSR (range −4 to + 53%) had a significant effect on rice yield over *beushening.* Similarly, PTR-M consistently increased rice yield by 16–26% over PTR-R, and by 5–23% over PTR-L. In the dry season, pulse crops (green gram and black gram) performed well compared to Indian mustard under rainfed cultivation. However, under irrigated conditions, dry-season rice yield was more productive than the rice equivalent yield of green gram, black gram and sunflower. We found that 1.03 M ha (i.e., ∼50%) of total rice-fallow areas of 2.1 M ha were suitable for growing short duration green gram and black gram in the dry season.

**Conclusions:**

We conclude that system productivity and cropping intensity can be increased by adoption of DSR and PTR-M in the wet season, and growing of green gram and black gram in the dry season.

**Implications:**

Odisha state can potentially produce an additional 0.67 million tonnes pulses if suitable rice-fallow areas are brought under green gram and black gram cultivation in the dry the season.

## Highlights

• Rice-based system productivity is poor due to low rice yield in the wet season and limited cropping in the dry season.
• Rice yield can be improved by adoption of direct seeded rice and mechanical transplanted rice
• Rice-fallow area in Odisha is 2.1 M ha, and 50% (1.03 M ha) of them are suitable for growing short duration crops.
• Pulses such as green gram and black gram can be grown in rice-fallow areas in rainfed condition.

## 1 Introduction

Rice (*Oryza sativa*) is consumed by more than half of the world’s population (Ciulu et al., 2018). India is the world’s second-leading producer of rice contributing 24% of global rice production (USDA, 2020). Food demand has increased over the years and will continue to increase, consequently putting food security in danger (OECD, 2015). Thus, India should focus on producing more food to meet its growing need by increasing rice yields in the wet season (monsoon season: June to October) and bringing more area (rice-fallow) under pulse or oilseeds cultivation in the dry season (post-monsoon season: November to April). Approximately 11.7 million hectares (M ha) of land in India are left fallow during the dry season after the rice harvest, which accounts for about 80% of the total rice-fallow area in South Asia (Gumma et al., 2016, NAAS, 2013). About 82% of India’s rice-fallow is concentrated in the eastern Indian states, namely Odisha, Chhattisgarh, Jharkhand, Assam, Bihar, eastern Uttar Pradesh, and West Bengal (Pande et al., 2012, Kumar et al., 2018a). Moreover, rice productivity in the wet season has also been stagnant for the past two decades in Eastern India (Mohanty and Yamano, 2017).

The prevalence of poverty in Odisha is high because of low agricultural growth (Hossain and Fischer, 1995) and frequent climatic extreme events-flooding, soil erosion, and drought (Government of Odisha, 2015, Bahinipati, 2014). Rice-based cropping systems are the dominant systems in Odisha where rice is cultivated on 3.6 M ha during the wet season, out of which 59% remains fallow in the dry season due to soil moisture stress (Das, 2012, Kumar et al., 2018a) and socio-economic constraints (Pande et al., 2000, Lal et al., 2017). Moreover, the average rice yield in this region is low compared to the national average because of in-season monsoon variability and sub-optimal crop management practices (Panneerselvam and Singh, 2019).

Traditionally, manual transplanting of rice seedlings in puddled fields (PTR-R) or *beushening* are the common methods of rice establishment in Odisha (Singh et al., 2020, Panneerselvam et al., 2020). Although the PTR-R has multiple benefits including weed suppression, optimum plant population, and nutrient availability (Johnson and Mortimer, 2005, Sharma et al., 2003), there are also numerous limitations associated with it. First, this method utilizes large quantities of water for crop establishment (Sudhir-Yadav et al., 2011, Chauhan and Opeña, 2012), labor (Singh and Sharma, 2012), and energy (Quilty et al., 2014), thus increasing the input cost. Next, puddling degrades soil structure (Alam et al., 2017), thereby often reducing the yield of the succeeding non-rice crop (Jat et al., 2022). Finally, the lack of availability of labor during transplanting and high labor costs reduce the profitability of rice farmers (Devkota et al., 2019, Sudhir-Yadav et al., 2017). *Beushening* is the broadcasting of ungerminated dry rice seeds at very high seed rates (100–120 kg ha^−1^) just before the onset of monsoon, followed by cross-plowing and weeding at 4–6 weeks after crop emergence when 10–15 cm of rainwater has accumulated in rice fields (Singh et al., 1994, Tomar, 2002). *Beushening* also has disadvantages such as low yield, high seed rate, and high labor and production costs (Panneerselvam et al., 2020). Nonetheless, *beushening* is widely practiced by resource-poor farmers in Eastern India because they obtain stable low yields under highly variable monsoon conditions, while limiting investments in crop production (Nayak and Lenka, 1988).

A large area of wet season rice in Odisha is left fallow in the dry season due to the lack of irrigation facilities, low residual soil moisture after rice harvest, lack of knowledge, lack of access to high-yielding varieties of short-duration pulses and oilseeds, animal grazing, and out-migration of labor during the dry season (Singh and Satapathy, 2019, Pasupalak et al., 2016, Abraham and Pingali, 2021). In addition, the growing long-duration rice varieties and the delayed procurement of rice at maturity, which leads to delays in vacating the field for the succeeding crop, plays a major role in the decision to leave fields fallow (Ranjan, 2016, Sarangi et al., 2019, Chandna and Mondal, 2020, Hoda et al., 2021). However, most rice-fallow areas have suitable climatic conditions to grow short-duration pulses and oilseeds (Chowdhury et al., 2020, NAAS, 2013). Pulses are ideal for the rice-fallow system since they require less water for cultivation and have a deep-rooted system to tap the available soil moisture up to 0.4 m of soil depth (Hazra and Bohra, 2021, Kumar et al., 2016, Liu et al., 2010). Pulses are efficient protein producers even under rainfed conditions with minimal inputs (Havlin et al., 2014, Adarsh et al., 2019). Moreover, pulses improve physical, chemical, and biological properties of the soil and reduce fertilizer requirement of the succeeding crops (Garg and Geetanjali, 2009). However, pulse production is uneconomical in the region due to lack of access to inputs (access to varieties, machines, irrigation, knowledge) and markets (Malo and Hore, 2020, Kumar et al., 2020). Pulses and oilseeds, however, have tremendous capacity to ensure sustainable intensification of rice-based cropping systems (Das et al., 2020).

The productivity of rice-based cropping systems in Odisha can be increased by mechanized crop establishment methods like DSR or PTR-M during the wet season. DSR with integrated weed management could be a potential alternative to PTR-R and *beushening* in Odisha (Panneerselvam et al., 2020, Susilawati et al., 2019). DSR saves 35–55% of irrigated water (Lav et al., 2007) and reduces labor requirements by 11–66%, depending on the region and season (Kumar et al., 2009; Rashid et al., 2009). Moreover, the adoption of DSR led to early harvest of rice by 7–10 days which enables the timely sowing of succeeding crops such as wheat, pulses and oilseeds (Jat et al., 2022). Together, these interventions may enhance the profitability of rice-based cropping systems in the region through greater cropping intensity and through increased productivity of the rice crop. Additionally, increasing pulse production may contribute to added protein consumption and dietary diversification.

There are knowledge gaps on (i) the performance of mechanized crop establishment methods in the wet season; (ii) suitable rice-fallow areas for pulses cultivation in the dry season and, (iii) performance of pulses in the rice-fallow areas. We hypothesized that mechanized crop establishment methods (DSR and PTR-M) will result in higher yield compared to traditional methods (PTR-R and *beushening)*. Furthermore, growing short duration pulses in the dry season will increase rice-based system productivity. To test the above hypotheses, four types of experiments were conducted in farmers’ fields in three districts between 2017 and 2019 with the following objectives: (1) to evaluate the performance of DSR compared to *beushening and* PTR-R; (2) to evaluate the performance PTR-M compared to PTR-R and PTR-L; (3) to identify rice-fallow areas suitable for pulses and oilseeds cultivation in the dry season; and (4) to evaluate the performance of short-duration pulses (*Vigna radiata*, *Vigna mungo*), and oilseeds (*Brassica. rapa* var. toria, *Helianthus annus*) in rice-fallow suitable areas.

## 2 Materials and methods

### 2.1 Study area

Three-years (2017–2019) of multi-location on-farm experiments were conducted in the districts of Bhadrak (21.0126° N, 86.6208° E), Cuttack (20.5168° N, 85.7256° E), and Mayurbhanj (22.0087° N, 86.4187° E) in Odisha, India. The major cropping system in all three districts is either rice-fallow or rice-pulses/oilseeds, though large areas remain fallow in the dry season. The climate of Bhadrak, Cuttack, and Mayurbhanj is subtropical with a mean annual rainfall of 1493, 1721, and 1596 mm respectively during the years 2017–2019. Around 92% of this rainfall was received from May to October during which the wet season rice is being grown (Supplementary fig. 1). The average annual maximum temperature remained between 33 °C and 35 °C and the average annual minimum temperature remained between 22 °C and 25 °C for all three districts during the study period (Supplementary fig. 2). The climate, soil properties, and other characteristics are summarized in Table 1. The soils are primarily red and laterite in the studied districts. The soil was acidic to alkaline (pH 4.3–8.0) in the Cuttack region, and acidic to strongly acidic in Bhadrak (pH 4.5–6.6) and Mayurbhanj (pH 4.5–5.5). This study was conducted under the CSISA project (www.csisa.org) districts: Bhadrak, Cuttack, and Mayurbhanj, where rice is the major crop in the wet season and large areas remain fallow in the dry season. These three districts, Bhadrak from North Eastern Coastal Plain, Cuttack from Mid Central Table Land, and Mayurbhanj from North Central Plateau represent three out of ten agro-climatic zones of Odisha, likely cover a wide range in eastern Indian states.

**Table 1:** Climate, soil, and other characteristics of three studied districts in Odisha (Das, 2012).

Characteristic	Mayurbhanj	Bhadrak	Cuttack
Agro-climatic zone	North Central plateau	North Eastern Coastal Plain Zone	East and southeastern coastal plain
Climate	Sub-tropical – hot and moist	Sub-tropical – hot and humid	Sub-tropical – hot and humid
Annual rainfall (mm)	1648	1568	1577
Monsoon rainfall (mm)	1361	1376	1467
Major cropping systems	Rice- fallow, Rice-pulses/oilseeds, Maize-fallow	Rice- fallow Rice-pulses/oilseeds	Rice- fallow Rice-pulses/oilseeds
Cropping intensity (%)	121	138	153
Kharif rice area (ha)	339000	165000	128000
Highland (%)	26	2	8
Medium Land (%)	37	38	40
Lowland (%)	37	60	52
Irrigated rice area in kharif season (%)	28	62	68
Major risks and uncertainties	Intermittent drought, lack of life-saving irrigation	Flood, cyclone, saline soil, submergence in lowland rice	Flood, cyclone, and submergence in lowland rice
Soil type	Laterite and red soil	Red and laterite, deltaic alluvium, coastal saline	Coastal saline, sandy, lateritic, alluvial, black and red
Soil texture	Sandy loam	Loam and clay loam	Coastal alluvial saline

### 2.2 Evaluation of direct seeded rice (DSR) and mechanical puddled transplanted rice (PTR-M) in the wet season

Two different sets of experiments were evaluated in the wet season. Experiment I was to evaluate DSR against manual random puddled transplanted rice (PTR-R) and *beushening*. Experiment II was to evaluate mechanical puddled transplanted rice (PTR-M) against PTR-R and manual line puddled transplanted rice (PTR-L).

Treatments in Experiment I:

*T1: Beushening*
*T2: Dry-direct seeded rice (DSR)*
*T3: Manual random puddled transplanted rice (PTR-R)*

Treatments in Experiment II:

*T1: Manual random puddled transplanted rice (PTR-R)*
*T2: Manual line puddled transplanted rice (PTR-L)*
*T3: Mechanical puddled transplanted Rice (PTR-M)*

The details of treatments mentioned above described in Table 2. Experiment I was conducted at 34 sites over three years: 15 sites in Bhadrak, 9 sites in Cuttack and 10 sites in Mayurbhanj. Experiment II was evaluated at 15 sites over two years in the wet season; 11 sites in Bhadrak, and 4 sites in Cuttack. All treatments were evaluated at each site. The size of the experimental plots for each treatment was 800–1000 m^2^. The treatments were not replicated at each farmer’s field; instead, individual farmer’s field were considered as a replication (Kottawa-Arachchi et al., 2022, Abeysiriwardena and Samita, 2016, Panneerselvam et al., 2020). The farmers and villages were selected in discussion with KVKs (Krishi Vigyan Kendra or Farm Science Center) and farmers who were willing to participate in the research were selected. Trials were conducted in farmer participatory research method and the inputs were supplied by the researchers, and applied by farmers in the presence of researchers or field technicians. Machines like seed drill and transplanter were brought by the field technicians to the experimental sites for sowing or transplanting.

**Table 2:** Treatment details of the wet season rice.

S.No	Treatment	Treatment details
**Experiment I**		
T1	*Beushening*	Broadcasting of ungerminated dry rice seeds @ 100–120 kg ha^−1^ in the month of June before the monsoon followed by wet cross-plowing, laddering and hand weeding at 30–40 days after sowing (DAS) when 10–15 cm of rainwater has accumulated in rice fields.
T2	Dry-direct seeded rice (DSR)	Sowing of ungerminated dry seeds @ 30 kg ha^−1^ with the use of seed drill on the same day of sowing of T1 in each site. Weeds were managed by the application of a tank-mix of bispyribac-sodium + pyrazosulfuron-ethyl @ 20 + 20 g ha^−1^ at 15–25 DAS as post-emergence, followed by one hand-weeding at 30–35 DAS. Herbicides were sprayed using a knapsack backpack sprayer fitted with a multi-flat nozzle boom in a spray volume of 500 litre water ha^−1^.
T3	Manual random puddled transplanted rice (PTR-R)	Sowing in rice nursery were taken up on almost the same day of sowing of T1 @ 50 kg ha^−1^ seed rate. Twenty-five to 30-day old seedlings (2–3 seedlings hill^−1^) were transplanted in the adjacent plot reserved for this treatment. Weeds were managed by the application of a tank-mix of bispyribac-sodium + pyrazosulfuron- ethyl @ 20 + 20 g ha^−1^ at 15–25 days after transplanting (DAT) as post-emergence, followed by one hand-weeding at 30–35 DAT.
**Experiment II**		
T1	Manual random puddled transplanted rice (PTR-R)	Same as T3 in Experiment I
T2	Manual line puddled transplanted rice (PTR-L)	Same as T3 in Experiment I except transplanted manually in line by using a rope. Row-row distance of 20 cm and plant-plant distance of 10 cm were maintained in LPTR.
T3	Mechanical puddled transplanted Rice (PTR-M)	15–18-day old seedlings raised through mat-type nursery were transplanted by mechanical transplanter at the spacing of 23 cm row to row and 14 cm plant to plant. In mat type nursery, pre-germinated seeds were broadcasted on a thin layer of soil and farmyard manure (FYM) mixture was placed on a perforated polythene sheet on the nursery bed. Mat-type nursery is a pre-requisite for transplanting mechanically as nurseries raised through conventional methods cannot be transplanted through the machine.

DAS- Days after sowing; DAT-Days after transplanting.

For Experiment I, land was cross-tilled by a four-wheel tractor with cultivator to a depth of about 15 cm before sowing. One light planking was used after sowing in T1 and T2 to crush the hard clods and compact the soil lightly to ensure that the seeds were covered with soil. This helps to avoid any phytotoxicity from pre-emergence herbicide*,* ensures better seed germination, and prevents moisture loss just after sowing. Rice variety Swarna was used in Mayurbhanj, and Swarna Sub-1 in Bhadrak and Cuttack. In Experiment I, sowing with was carried out with use of seed drill in DSR (T2) on the same day as broadcasting of seeds under *beushening* (T1) and establishing the rice nursery under PTR-R (T3). For Experiment II, Swarna Sub-1 rice variety was used in both districts. Rice seeds were soaked in a water mixture with Carbendazim 50% WP @ 2 g kg^−1^ seed for 10–12 h to control seed and soil borne diseases before sowing into the nursery bed. A seed rate of 50 kg ha^−1^ was used in PTR-R and PTR-L, and 38–45 kg ha^−1^ in PTR-M.

Fertilizer rates (kg ha^−1^), namely N80: P40: K40: Zn5, were the same for all treatments in Experiment I and II. However, the timing of fertilizer application varied among the treatments in Experiment I. For T1 in Experiment I, at sowing, 33% of N and 100% P as diammonium phosphate (DAP), 100% K as muriate of potash (MOP) and 100% Zn as Zinc sulphate heptahydrate (ZnSO_4_0.7 H_2_O) were applied. The remaining N in the form of urea was broadcasted in two equal splits: the first top dressing at the time of the *beushening* operation (30–40 DAS, dependent on the rainfall) and the second top dressing at panicle initiation (50–60 DAS). In T2, fertilizer application was the same as that of T1 except the first top dressing of N was done at 25–30 DAS after post-emergence herbicide application at 20–25 DAS. In T3, 33% N as DAP, 100% P as DAP, 50% of K as MOP and 100% Zn as Zinc sulphate heptahydrate (ZnSO_4_0.7 H_2_O) were applied at transplanting. The remaining N as urea was applied in two equal splits at active tillering (20–25 DAS) and panicle initiation (50–60 DAS), and the remaining 50% of K was applied at panicle initiation.

Fertilizer rates and timing for all three treatments in Experiment II were similar to T3 of Experiment I. In all three treatments, pre-emergence herbicide pretilachlor with safener 30.7% EC @ 500 g ha^−1^ was applied within 3 days of transplanting. Post-emergence herbicide tank mixture of bispyribac-sodium 10% SL @ 20 g ha^−1^ + pyrazosulfuron-ethyl 10% WP @ 20 g ha^−1^ was applied 15–20 DAT and one spot hand weeding was done at 30–35 DAT.

### 2.3 Identify rice-fallow areas suitable for pulses and oilseeds cultivation in the dry season

An approach of combining multi-temporal Earth Observation (EO) data from Landsat-8 Operational Land Imager (OLI) and Sentinel-1satellite sensors from 2018 to 2021 was adopted to identify rice-fallow lands in Odisha. Three different levels of classification for agriculture areas were adopted. The first-level classification was the overall agriculture area. In the second-level, four seasonal class combinations were classified and defined as: (a) Kharif (wet season)-Rabi (dry season), (b) Kharif-Fallow, (c) Fallow-Rabi, and (d) Fallow-Fallow. The third-level classification was specifically focused on rice crop and therefore, four rice-based cropping system classes were used, namely: (a) Rice-Rice (i.e., rice during both seasons), (b) Rice-Rabi (Rice during Kharif season and other crops during Rabi season), (c) Rice-Fallow (i.e., Rice during Kharif season and Fallow during Rabi season), and (d) Fallow-Rice (i.e., Fallow during Kharif season and Rice during Rabi season).

For the first-level classification, Principal Component Analysis (PCA) was utilized for creating segments and separations of different vegetation classes. PCA is particularly effective for identifying the different phenological patterns and intra-seasonal variability in a time series image. For the second-level classification, the unsupervised classification technique with the k-means algorithm was applied to map seasonal cropping systems. For the third-level classification, the rice crop areas during the monsoon season were extracted from the time-series of Sentinel-1 data. The rice crop exhibits variable phenological response of SAR backscattering coefficient in decibels (dB) in different growing stages of crop. The dB value remains very low during transplantation time due to flooding conditions in puddled fields and it increases with the increasing growth of rice plants until the middle stage or early maturing stage. These phenological responses through time are unique in dB compared with other crops and therefore, the phenological parameters extracted from the time series dB profile were utilized in delineating the rice crop (Nguyen et al., 2016).

The daily SMAP (Soil Moisture Active Passive) L-band soil moisture data from October to February for 2018–2021 years were obtained from the National Snow and Ice Data Center Distributed Active Archive Center (NSIDC DAAC) of NASA (National Aeronautics and Space Administration). NASA’s SMAP mission provides satellite estimates for both surface soil moisture (0–5 cm) and root zone soil moisture (0–100 cm) data at a resolution of 9 km. In our study, we used the surface soil moisture (0–5 cm) depth product in rice-fallow areas. We derived the suitability classes by exploring the spatio-temporal soil moisture profiles with optimum levels (0.3 m^3^/m^3^) of soil moisture availability during the fallow period (October to February) after the Kharif rice harvest across different locations. Based on the sowing window during the season combined with duration of the optimum soil moisture availability, suitable areas were identified from November to January for targeting short-duration and water-efficient pulse crops. We classified these zones into four categories: suitable, moderately suitable, marginally suitable, and not suitable. Those areas where optimum soil moisture was available until the first week of February were considered suitable, while areas with availability up to the end of January were moderately suitable, areas with availability until mid-January were marginally suitable, and areas with no residual soil moisture were classified as not suitable.

### 2.4 Evaluation of short duration pulses and oilseeds in suitable rice-fallow areas in the dry season

Two different sets of experiments were evaluated in rice-fallow areas in the dry season. First set was to evaluate short duration pulses and oilseeds in rice-fallow areas using residual soil moisture under rainfed conditions (Experiment III); a second set was to evaluate four different crops under irrigated situation (Experiment IV).

The treatments in Experiment III under rainfed conditions were:

*T1: Green gram (Vigna radiata,* variety *IPM-2–14)*
*T2: Indian mustard (Brassica rapa,* var*. toria,* variety *Uttara)*
*T3: Black gram (Vigna mungo,* variety *PU-35)*

And the treatments in Experiment IV under irrigated conditions were:

*T1: Rice (*variety *Bina Dhan11)*
*T2: Indian mustard-Toria (*variety *M-27)*
*T3: Green gram (*variety *IPM-2–14)*
*T4: Sunflower (*variety *MSFH-17).*

Each treatment was replicated at five farmers’ fields in each district. After wet season rice harvesting, 2–3 tillage operations with a cultivator followed by planking were completed before sowing of green gram, black gram, sunflower and Indian mustard-toria. In the case of rice, irrigation was applied in the field and puddling was done 2–3 days before transplanting. Manual puddled line transplanting (PTR-L) was followed for rice, so, inputs and cultivation practices were the same as mentioned in T2 (PTR-L) of Experiment II in the wet season.

Seeds were sown using a seed drill at a seed rate of 20 kg ha^−1^ for green gram and black gram, and 5 kg ha^−1^ for Indian mustard-toria and sunflower. Sowing was performed from the end of November to the first week of December and harvesting was done from the second to the third week of February. For Experiment III under rainfed conditions, fertilizer rates were N12: P 25: K 10 kg ha^−1^ for green gram, and black gram, and N50: P: K25 kg ha^−1^ for Indian mustard-toria were applied in the form of DAP and MOP at the time of sowing. The fertilizer rate increased for irrigated conditions and rates were N 20: P40: K20 kg ha^−1^ for green gram and N 60: P30: K30 kg ha^−1^ for Indian mustard-toria in the form of DAP and MOP at the time of sowing. For sunflower the fertilizer rate was N 60: P 80: K60 kg ha^−1^. Sulphur fertilizer was applied @ 25 kg ha^−1^ at the time of sowing of Indian mustard-toria. To control weeds in both rainfed and irrigated conditions, quizalofop-ethyl (Turga super 5% EC) @ 37.5 g ha^−1^ was applied at 20–25 DAS in green gram, black gram and Indian mustard-toria fields. In sunflower fields, fluchloralin (Basalin 45% EC) @ 1 kg ha^−1^ was applied at 15 DAS followed by manual earthing up at 30 DAS.

### 2.5 Crop harvest and yield estimation

At physiological maturity, rice was harvested from three randomly selected spots of 5 m^2^ from each treatment. Harvested rice was threshed manually and grain moisture content determined with a moisture meter. Grain yield was expressed at 14% moisture content. Similarly, non-rice crops were harvested from a specified unit area (10 m^2^) from each field and recorded. Rice equivalent yield (REY) for non-rice crops was calculated using the formula:

REY = Yx (Px/Pr),

where Yx is the non-rice crop yield (t ha^−1^), Px is the price of non-rice crops (US$ kg^−1^) and Pr is the price of rice (US$ kg^−1^). The minimum support price (fixed by Government of India) was used for calculating REY.

### 2.6 Statistical analysis

Analysis of variance (ANOVA) in the R programming environment (version 3.6.1) was used to analyze the data and the LSD was used at a P-value ≤ 0.05 to compare the differences among treatment means. Moreover, to interpret the mathematical relationship (strength and character) between variables, regression analysis was performed.

## 3 Results

### 3.1 Evaluation of direct seeded rice (DSR) and mechanical puddled transplanted rice (PTR-M) in the wet season

Two different sets of experiments were evaluated in the wet season. First set was to evaluate DSR against PTR-R and *beushening* (Experiment I). In Experiment I, rice yield was higher in the Bhadrak district compared to the Mayurbhanj and Cuttack districts (Table 3). Across years and districts, the highest grain yield was obtained with DSR followed by PTR-R and *beushening*. The yield increase in DSR over *beushening* was 0.32–0.77 t ha^−1^ (6–15%) in Bhadrak, and 1.70–1.78 t ha^−1^ (52–53%) in Mayurbhanj. Similarly, yield increase in DSR over PTR-R was 0.18–0.59 t ha^−1^ (3–11%) in Bhadrak, and 0.33–0.49 t ha^−1^ (7–10%) in Mayurbhanj. PTR-R produced higher grain yield in all the districts over *beushening* and significantly higher yields in Mayurbhanj, in Bhadrak in 2019 and in Cuttack in 2018. Overall, out of seven (districts x years) trials (Table 3), the yield in DSR was significantly higher than *beushening* methods and PTR-R in four trials and on-par with remaining three trials.

**Table 3:** Grain yield (t ha^−1^) under different crop establishment methods in rice during the *kharif* season (Experiment I).

Treatment	Bhadrak			Cuttack		Mayurbhanj	
	2017	2018	2019	2018	2019	2017	2018
T1: Manual broadcast fb *beushening (Beushening)*	4.97 ± 0.36^b^	5.30 ± 0.33^a^	5.21 ± 0.11^c^	2.98 ± 0.17^b^	5.89 ± 0.17^a^	3.34 ± 0.17^c^	3.26 ± 0.13^c^
T2: Dry direct seeded rice (DSR)	5.74 ± 0.48^a^	5.15 ± 0.48^a^	5.53 ± 0.08^a^	3.76 ± 0.67^a^	5.67 ± 0.72^a^	5.12 ± 0.15^a^	4.96 ± 0.12^a^
T3: Manual random puddled transplanted rice (PTR-R)	5.15 ± 0.27^b^	5.52 ± 0.35^a^	5.35 ± 0.08^b^	4.04 ± 0.59^a^	5.91 ± 0.21^a^	4.63 ± 0.08^b^	4.63 ± 0.16^b^

Mean of rice yield ± SD within a column followed by the same letter is not statistically different using the LSD test at P-value ≤ 0.05.

**Fig. 1:**
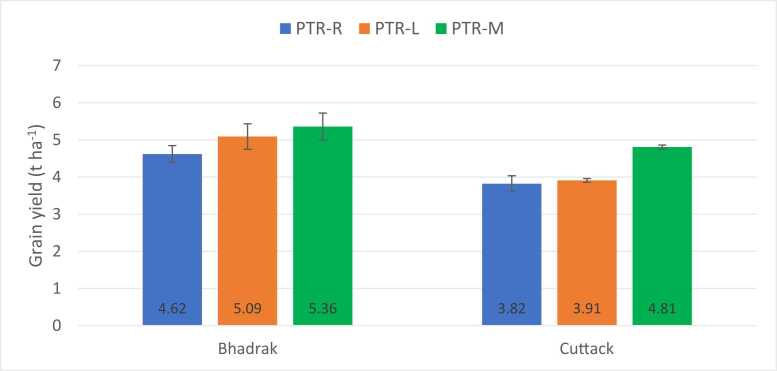
Grain yield (t ha^−1^) under different crop establishment methods in rice (Experiment – II). Note: Two years of on-farm trials combined due to no significant differences in the year. PTR-R: Manual random puddled transplanted rice; PTR-L: Manual line puddled transplanted rice; PTR-M: Mechanical puddled transplanted rice.

A second set of experiments was designed to evaluate mechanical puddled transplanted rice (PTR-M) against manual random puddled transplanted rice (PTR-R) and manual line puddled transplanted rice (PTR-L) (Experiment II). The maximum yield of 5.36 t ha^−1^ was obtained in Bhadrak and 4.81 t ha^−1^ in Cuttack under PTR-M followed by PTR-L and PTR-R (Fig. 1). The corresponding yield increase in PTR-M over PTR-R was 0.74 t ha^−1^ (16%) in Bhadrak and 1.00 t ha^−1^ (26%) in Cuttack (Fig. 1). Similarly, the yield increase in PTR-M over PTR-L was 0.27 t ha^−1^ (5.3%) in Bhadrak and 0.9 t ha^−1^ (23%) in Cuttack. Our results suggest that both DSR and PTR-M have a significant effect on rice yields in this region.

### 3.2 Identify rice-fallow areas suitable for pulses and oilseeds cultivation in the dry season

**Fig. 2:**
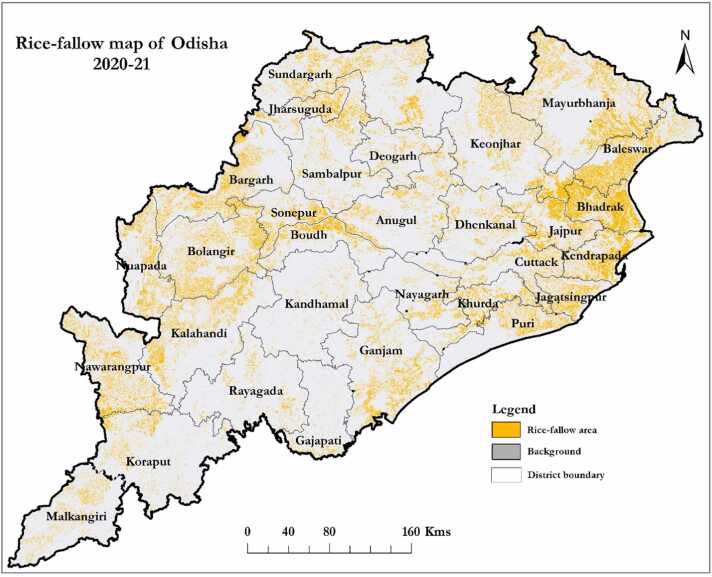
Rice-fallow area in Odisha.

**Fig. 3:**
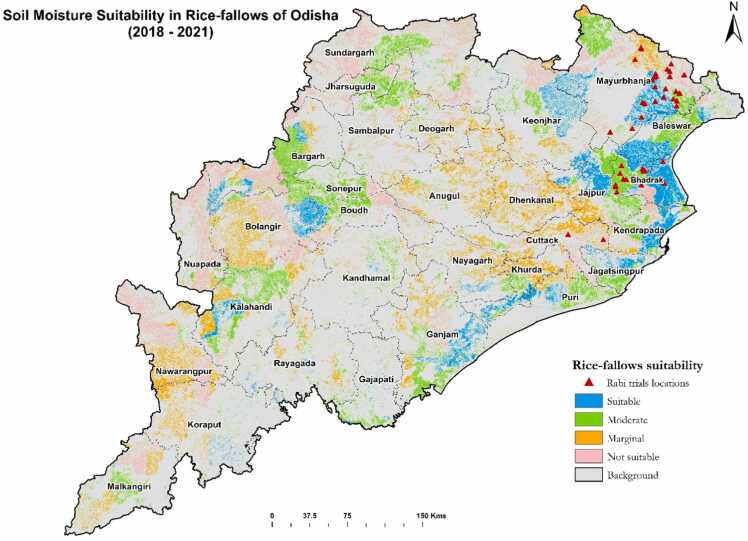
Spatial distribution of soil moisture suitability during 2018–2021 for growing dry season crops.

The area under rice, rice-fallow and their temporal dynamics between 2018 and 2021 were mapped using temporal Sentinel-1 SAR and Landsat-8 OLI EO data. The area under rice in the wet season, the rice-fallow area in the dry season and the rice-fallow area suitable for growing short duration pulses and oilseeds are presented district-wise in Table 4 and Fig. 2. The average rice area in Odisha for the years 2018–2021 was 3.57 M ha with an overall classification accuracy of 93% (Table 4). The declining residual soil moisture over time is the major challenge to growing short duration pulses (55–60 days) and oilseeds in rainfed rice-fallow areas. The 3-day composite imageries of SMAP data were analyzed from October to February (2018–2021) for each year to generate the soil moisture suitability maps. A threshold value of 30% field capacity was considered for each pixel of the temporal stacked soil moisture data (Hanson et al., 2000). A map with three-year average soil moisture to target dry season crops (especially short-duration and water-efficient pulses) is depicted in Fig. 3. We analyzed and mapped the trends of rice fallows over the years 2018–2021 and estimated that there were 2.1 M ha rice fallow areas in Odisha, out of which 1.03 M ha (49% of total rice fallows) were considered as suitable for growing short duration pulses or oilseeds using residual soil moisture under rainfed conditions (Table 4). It is estimated that about 176,964 ha area in the Mayurbhanj district remains rice-fallow after rice harvest, of which around 110,274 ha (62% of rice fallow area) is suitable for growing short duration pulses and oilseeds (Table 4). Similarly, 128,885 ha in Bhadrak and 52,551 ha in Cuttack remains rice-fallow after rice harvest, of which 81% and 52% of rice fallow area are suitable for short duration pulses and oilseeds respectively.

**Table 4:** Area under rice, rice-fallow and suitable area for cropping in the dry-season in Odisha (2020–2021).

S. No	District	Kharif 2020 (ha)	Rice-fallow area		Rice-fallow area suitable for cropping	
			ha	%	(ha)	**%**
1	Angul	82,233	62,212	76	43,042	69
2	Baleshwar	1,92,645	1,20,529	63	91,591	76
3	Bargarh	2,29,090	1,03,698	45	42,919	41
4	Bhadrak	1,55,790	1,28,885	83	1,03,894	81
5	Bolangir	1,99,990	1,35,901	68	19,594	14
6	Boudh	56,203	44,782	80	30,783	69
7	Cuttack	1,13,960	52,551	46	27,574	52
8	Deogarh	38,934	22,851	59	14,092	62
9	Dhenkanal	92,396	44,962	49	19,179	43
10	Gajapati	27,168	13,166	48	8719	66
11	Ganjam	2,07,182	99,660	48	61,493	62
12	Jagatsinghpur	81,108	44,584	55	32,824	74
13	Jajpur	1,12,492	68,825	61	53,635	78
14	Jharsuguda	51,077	39,341	77	11,578	29
15	Kalahandi	1,89,616	99,081	52	18,423	19
16	Khandamal	40,170	17,843	44	13,346	75
17	Kendrapada	1,22,102	81,277	67	77,006	95
18	Kendujhar	1,73,013	1,09,182	63	34,631	32
19	Khordha	85,693	48,537	57	29,163	60
20	Koraput	1,00,624	48,203	48	2734	6
21	Malkangiri	80,725	41,991	52	7157	17
22	Mayurbhanj	2,83,569	1,76,964	62	1,10,274	62
23	Nabarangapur	1,17,128	1,01,492	87	6379	6
24	Nayagarh	72,692	32,066	44	14,191	44
25	Nuapada	90,260	67,372	75	6752	10
26	Puri	95,288	47,350	50	35,833	76
27	Rayagada	56,808	26,900	47	3645	14
28	Sambalpur	1,29,241	58,011	45	42,834	74
29	Sonepur	1,01,629	47,057	46	32,109	68
30	Sundargarh	1,92,719	1,10,323	57	36,360	33
	**State**	**35,71,545**	**20,95,597**	**59**	**10,31,754**	**49**

### 3.3 Evaluation of short duration pulse and oilseeds in suitable rice-fallow areas in the dry season

Two sets of experiments were evaluated in rice-fallow areas in the dry season. First set was to evaluate short duration green gram, black gram, and Indian mustard-toria in rice-fallow areas using residual soil moisture under rainfed conditions (Experiment III). Non-rice crop yields were converted into rice equivalent yield (REY) as explained in Materials and Methods for comparison. During 2017 and 2018, green gram had a significantly higher REY than black gram and Indian mustard-toria in Mayurbhanj (Table 5). The REY of green gram was higher by 0.50–0.83 t ha^−1^ (36–55%) over black gram, and 1.10–1.50 t ha^−1^ (146–178%) over Indian mustard-toria in Mayurbhanj during 2017 and 2018 (Table 5). Whereas in 2019, in both Cuttack and Mayurbhanj regions, black gram had a significantly higher REY than green gram and Indian mustard-toria. Black gram had a higher REY of 1.5 t ha^−1^ (80%) in Mayurbhanj and 0.61 t ha^−1^ (23%) in Cuttack than green gram. Similarly, the REY of black gram was much higher, i.e., 2.34 t ha^−1^ (225%) more in Mayurbhanj, and 1.21 t ha^−1^ (60%) more in Cuttack in comparison to Indian mustard-toria.

**Table 5:** Actual crop yield (t ha^−1^) and rice equivalent yield (t ha^−1^) (REY) of crops grown in rice-fallow in the dry season under rainfed conditions (Experiment III).

Treatment	Mayurbhanj						Cuttack	
	2017		2018		2019		2019	
	Actual yield	REY	Actual yield	REY	Actual yield	REY	Actual yield	REY
T1:Green gram	0.50	1.90 ± 0.13^a^	0.60	2.33 ± 0.14^a^	0.60	1.87 ± 0.06^b^	0.86	2.63 ± 0.15^b^
T2:Toria	0.30	0.77 ± 0.03^c^	0.30	0.84 ± 0.02^c^	0.37	1.04 ± 0.09^c^	0.73	2.03 ± 0.21^c^
T3:Black gram	0.30	1.40 ± 0.03^b^	0.30	1.51 ± 0.04^b^	1.0	3.38 ± 0.48^a^	1.0	3.24 ± 0.17^a^

REY- rice equivalent yield

Mean of rice equivalent yield ± SD within a column followed by the same letter is not statistically different using the *LSD test* at P-value ≤ 0.05.

A second set was to evaluate rice and non-rice crops (Indian mustard-toria, green gram, and sunflower) under irrigated conditions or where one to two supplemental irrigations were available (Experiment IV). The results show that rice in the dry season performed better than non-rice crops (Table 6). Of the non-rice crops, sunflower (T4) had the highest REY followed by green gram (T3) compared to Indian mustard-toria (T2) (Table 6). Rice yield was higher by 0.95 t ha^−1^ (33%), 3.2 t ha^−1^ (111%), and 2.18–2.52 t ha^−1^ (61–66%) over REY of green gram in Mayurbhanj, Cuttack, and Bhadrak, respectively. Similarly, rice yield was higher than the REY of Indian mustard-toria by 2.74 t ha^−1^ (245%), 3.98 t ha^−1^ (190%), and 3.16–3.61 t ha^−1^ (123–133%) in Mayurbhanj, Cuttack, and Bhadrak, respectively. Among all the non-rice crops, Indian mustard-toria recorded significantly lowest REY in all three districts both in rainfed and irrigated conditions.

**Table 6:** Actual crop yield (t ha^−1^) and rice equivalent yield (t ha^−1^) of crops grown in the dry season under irrigated conditions *(Experiment – IV)*.

Treatment	Mayurbhanj		Cuttack		Bhadrak			
	2019		2019		2018		2019	
	Actual yield	REY	Actual yield	REY	Actual yield	REY	Actual yield	REY
T1: Rice	3.85	3.85 ± 0.22^a^	6.07	6.07 ± 0.12^a^	5.73	5.73 ± 0.40^a^	6.33	6.33 ± 0.09^a^
T2: Toria	0.40	1.12 ± 0.06^c^	0.75	2.09 ± 0.19^c^	1.0	2.57 ± 0.18^d^	0.97	2.72 ± 0.05^d^
T3:Green gram	0.95	2.90 ± 0.07^b^	0.94	2.87 ± 0.05^b^	0.95	3.55 ± 0.25^c^	1.0	3.81 ± 0.16^c^
T4: Sunflower	-	-	-	-	1.88	5.31 ± 0.18^b^	1.65	4.84 ± 0.93^b^

REY- Rice equivalent yield

Mean of rice equivalent yield ± SD within a column followed by the same letter is not statistically different using the *LSD test* at P-value ≤ 0.05.

## 4 Discussion

Cropping intensity and system productivity is low in Odisha due to 59% of wet season rice areas being left fallow in the dry season (Table 4), and the lack of adoption of mechanization in rice cultivation that could raise attainable yield in the wet season (Panneerselvam et al., 2020). The average rice yield in the wet season in Odisha is lower than in the neighboring states and this is associated with the widespread use of traditional rice crop establishment methods such as *beushening* and manual transplanting with older seedlings (Tomar, 2002, Uphoff, 2002, Das, 2012). To improve the cropping intensity and system productivity of rice-based systems in Odisha, farmers and extension need to consider the system as a whole, and to recognize that decisions in the rice phase can both raise rice yields and increase the options for using the large areas of fallow in the dry season. There are several possible interventions in the rice phase, such as timely rice establishment with DSR (McDonald et al., 2022, Panneerselvam et al., 2020). Additionally, the adoption of DSR will result in the earlier harvest of the wet season rice compared to transplanted rice because of the absence of transplanting shock under DSR, which results in earlier maturity by 7–10 days (Parthasarathi et al., 2012). This offers farmers the opportunity to grow pulses or oilseeds as a succeeding crop in rice-fallow areas with effective use of residual soil moisture (McDonald et al., 2022). Our study shows that system productivity can be improved by increasing the productivity of rice yield through adoption of DSR and PTR-M in the wet season and cultivation of green gram or black gram in suitable rice-fallow areas.

Our results showed that the DSR produced higher yields than *beushening* and PTR-R in four out of seven site-years (Experiment I). The first and foremost reason for this increase could be due to the uniform crop emergence and better weed management in DSR compared to *beushening*. In *beushening*, seeds are broadcast by hand followed by a tillage operation which leads to uneven crop emergence with some seeds going deep in the soil and some in the soil surface. Early weed competition is also relatively high in *beushening* since the weeds are not controlled until the *beushening* operation is carried out 30–40 DAS when 15–20 cm of rainfall is received (Tomar, 2002). Under DSR, effective weed management practices were performed using herbicides in a timely manner (Panneerselvam et al., 2020). Another possible reason for the increased yield in DSR could be due to the higher recovery efficiency of applied fertilizers and less weed competition (Sheela and Alexander, 1995). On the other hand, no significant yield differences were observed among the treatments in 2018 in Bhadrak and Cuttack. One possible reason could be that rains were favourable in the wet season in 2018 (Supplementary fig. 1) such that the *beushening* and other crop management practices were timelier (earlier) in both *beushening* and manual transplanting methods. Another reason could be due to access to irrigation (Table 1) and favorable soil type and hydrology (i.e., poorly drained soils) that favor longer stagnation of water and hence better weed control and higher nutrient availability compared to other two districts (Panneerselvam et al., 2020). However, it is hard to predict the amount and distribution of rainfall every year, in addition to the shortage of labor prevalent in this region for manual transplanting and *beushening* operations. Thus, DSR can be a potential alternative to produce a higher yield in the wet season with lesser inputs of seeds, water, and labor.

Our results also showed that the PTR-M produced higher yields than the PTR-R and PTR-L (Fig. 1, Experiment II). The higher rice yield in PTR-M could be attributed to the use of young seedlings (Uphoff, 2002). For instance, under manual transplanting, 25–30-day old seedlings were used in our study, whereas for PTR-M, 15–18-day old seedlings were transplanted which might have resulted in the early adaptation of the seedlings. Moreover, seedlings in the mat-type nursery used for PTR-M have less damaged roots resulting in less transplanting shock, which is a major problem in the PTR-R and PTR-L. Additionally, along with the improved yield, PTR-M allows more timely planting and reduces the production cost by reducing the labor cost for transplanting (Goswami et al., 2020). In summary, both DSR and PTR-M, apart from producing higher yields, also address labor scarcity along with decreased input costs (FAO, FAOSTAT, 2017; Devkota et al., 2020; Pathak et al., 2013). However, there are also major challenges in the adoption of DSR and PTR-M due to the lack of awareness of the technology, the high cost of machines, inadequate availability of mat-type nursery, and lack of skilled workers (Sudhir-Yadav et al., 2017). To encourage farmers to adopt mechanization, the Indian government is subsidizing the cost of transplanter and seed drill machines (CSISA, 2017). However, other strategies such as capacity building of famers and extension agents, creating machine service providers, improvement in seed drill for uniform plant to plant distance within the row, and strengthening public-private partnership on market development of quality seed drills are important for scaling mechanization in crop establishment methods.

Earth observation data, including SMAP soil moisture data, facilitates the precise targeting of suitable areas for cropping pulses and oilseeds in the dry season. This approach can facilitate improved rice-fallow system productivity and income of farmers through precise targeting of technology by bringing potential rice-fallows area into cultivation. Our results showed that green gram and black gram can be grown successfully in 81%, 52%, and 62% of the rice-fallow areas of Bhadrak, Cuttack and Mayurbhanj districts, respectively (Table 4). However, Indian mustard-toria, has less potential compared to pulses due to low yield (Table 5). The proper selection of short duration pulses and oilseeds is important to utilize the available soil moisture in rice-fallow areas (Das et al., 2014, Singh and Satapathy, 2019, Abraham and Pingali, 2021).

Next, we analyzed the performance of rice, pulses, and oilseeds during the dry season under irrigated conditions or areas where supplemental irrigation is available. As expected, rice yield is significantly higher followed by sunflower and green gram (Table 6). Although enhancing productivity is important, protecting the environment and the sustainable use of natural resources are also highly crucial. Research has shown that the continuous cultivation of rice can lead to the depletion of soil nutrients (NAAS, 2013) and an increase in greenhouse gases such as methane and nitrous oxide emissions (Kritee et al., 2018). The REY of toria was lowest among the crops which could be due to very short winter period in Odisha, heat stress during flowering or maturity and less market price. Although sunflower yield was higher than green gram, growing pulses in rice-fallow can be beneficial in two ways. First, green gram is a short duration pulse and takes 60–65 days to mature (Government of India, 2020), whereas sunflower matures in 85–88 days (Mahapatra et al., 2021). Therefore, cropland is readily available to cultivate a summer crop if irrigation is available. Another reason to include pulses in the rice-fallow system is to improve the soil nitrogen which can reduce nitrogen requirement of the succeeding crops (O’donovan et al., 2014, Parton and Rasmussen, 1994, Ganeshamurthy, 2009).

This study showed that rice-based cropping system productivity in Odisha can be improved by adoption of mechanized crop establishment methods (DSR and PTR-M) in the wet season and growing of green gram and black gram (pulses) in suitable rice fallow areas. The average yield of pulses from fallow areas across districts and years was 0.65 t ha^−1^ (Table 5) and we also found that 49% of rice-fallow areas (1.03 M ha) are suitable for cropping in the dry season (Table 4). If this suitable area is brought under pulse cultivation, then Odisha state can potentially have an additional production of 0.67 million tonnes of pulses from the dry season. The suitability map can be utilized for further validation and demonstration of short duration pulses in other districts and can be used for bringing more rice-fallow areas under cropping in Odisha. In parallel, programs will be needed to share knowledge, increase the availability of machinery, and support marketing. Also, promoting DSR in the wet season will result in the earlier harvest of rice by 7–10 days (Parthasarathi et al., 2012) which facilitates early sowing of pulses with residual soil moisture. The promotion of high yielding seed materials, mechanization for timely sowing of pulses, and incentives for pulses cultivation can bring more areas in cropping and thereby increase the cropping intensity and systems productivity.

## 5 Conclusion

Our study suggests that mechanized crop establishment methods (DSR and PTR-M) increased rice yield compared to traditional crop establishment methods (*beushening* and PTR-R). The yield gain in DSR is 0.32–1.78 t ha^−1^ over *beushening,* and 0.18–0.59 t ha^−1^ over PTR-R across three districts. Similarly, yield gain in PTR-M is 0.74–1.00 t ha^−1^ over PTR-R and 0.27–0.9 t ha^−1^ over PTR-L across the districts. In this study, we estimated that 49% of total rice-fallow area (2.1 M ha) of Odisha is suitable for cultivation of short duration pulses and oilseeds in the dry season. Green gram and black gram produced 0.5–1.0 t ha^−1^ in the rice-fallow areas under rainfed conditions across site-years suggesting an additional pulse production of 0.5–1.0 million tons from 1.03 M ha of suitable rice-fallow areas. Further studies are required to validate the suitability map and demonstrate the pulses in other districts in rice-fallow areas. However, a proper government policy is required promote mechanization including capacity building of famers and extension agents, creating machine service providers, strengthening public-private partnership on market development of quality seed drills. Also, promoting mechanization for timely sowing of pulses, and incentives for pulses cultivation can bring more rice-fallow areas into the cropping and thereby increase the cropping intensity and systems productivity.

## Declaration of Competing Interest

The authors declare that they have no known competing financial interests or personal relationships that could have appeared to influence the work reported in this paper.

## Data Availability

Data will be made available on request.
