# Supplementary material for: Crop establishment and diversification strategies for intensification of rice-based cropping systems in rice-fallow areas in Odisha
Source: Field Crops Res. 2023 Oct 15;302:109078. doi: 10.1016/j.fcr.2023.109078 (PMC10565835; doi:10.1016/j.fcr.2023.109078)
Supplement: Supplementary file 1 — Supplementary material. [file mmc1.docx]

**Supplementary file:**

**Supplementary fig. 1. Monthly rainfall during the rice-growing period in 2017-2019 for Bhadrak, Cuttack, and Mayurbhanj districts**

The monsoon season is from May to October during which the wet season rice is grown. Very little rainfall was received during the dry season (November to April).

**Supplementary fig.2. Monthly average maximum and minimum temperatures for the Bhadrak, Cuttack, and Mayurbhanj districts during 2017-2019**

Max -Maximum; Min- Minimum
